# Autoimmune sensorineural hearing loss as presenting manifestation of paediatric Behçet disease responding to adalimumab: a case report

**DOI:** 10.1186/s13052-016-0291-2

**Published:** 2016-09-06

**Authors:** Manuela Marsili, Valentina Marzetti, Marta Lucantoni, Giuseppe Lapergola, Marco Gattorno, Francesco Chiarelli, Luciana Breda

**Affiliations:** 1 Department of Pediatrics, University of Chieti, Via dei Vestini 5, Chieti, 66100 Italy; 2 UO Pediatria II, G. Gaslini Scientific Institute, Genova, Italy

**Keywords:** Behçet disease, Vasculitis, Pediatrics, Children, Treatment, Adalimumab

## Abstract

**Background:**

Autoimmune sensorineural hearing loss, also known as autoimmune inner ear disease (AIED) is a rare clinical entity characterized by progressive and bilateral sensorineural hearing loss often accompanied by vestibular symptoms. Diagnosis is essential as a consistent number of patients show a positive response to steroids alone or in association with other immunosuppressive drugs. AIED is defined as primary when the disease is limited to the ear, whereas in up to a third of cases it is associated to other systemic autoimmune diseases such as Behçet disease (BD).

BD is a rare multisystem vasculitis characterized by recurrent oral and genital aphtosis, uveitis, skin lesions, neurological and vascular manifestations. Clinical presentation is variable thus making the diagnosis difficult in many instances. The choice of therapy is also limited by the scarceness of high-quality therapy studies.

**Case presentation:**

We present a 15-year-old-boy with six months of history of fever, dizziness, tinnitus and ataxia. He had a final diagnosis of AIED associated to BD and was successfully treated with the anti-tumor necrosis factor (TNF)-α adalimumab.

**Conclusions:**

This case report points out to the diagnostic and therapeutic challenges of BD especially when unusual symptoms are the prominent manifestations of the disease. It also suggests that adalimumab is a good therapeutic option in children with BD and audiovestibular symptoms.

## Background

Autoimmune sensorineural hearing loss, also known as autoimmune inner ear disease (AIED) results from dysfunction of the inner ear, the vestibule-cochlear nerve or the brain processing centres [1]. In most cases AIED is idiopathic and involves only the ear, however, in about a third of cases, it occurs in the context of systemic autoimmune diseases such as Cogan’s syndrome, Wegener’s granulomatosis, sarcoidosis, juvenile idiopathic arthritis and Behçet disease (BD) (Table 1) [1, 2]. Treatment usually includes corticosteroids and immunosuppressive drugs but refractory cases are described [1].

**Table 1:** Systemic autoimmune diseases associated with audio-vestibular manifestations

Disease	
Vogt-Koyanagi-Harada syndrome Cogan’s syndrome Systemic lupus erythematosus Systemic vasculitis Rheumatoid arthritis Juvenile Idiopathic Arthritis Behçet disease Sarcoidosis Panarteritis nodosa Wegener’s granulomatosis Hashimoto thyroiditis Sjögren’s syndrome Systemic sclerosis Crohn’s disease Ulcerative colitis Ankylosing spondylitis Multiple sclerosis	

Adapted from Rheumatology 2013;52:780-89

BD is a rare multisystem vasculitis characterized by recurrent oral and genital aphtosis, uveitis, skin lesions, neurological and vascular manifestations [3]. BD has an endemic distribution along the ancient Silk Road, but is rare in Europe and in the U.S.A [4]. Clinical presentation is variable thus making the diagnosis difficult in many instances. A correlation between genetic predisposition and triggering extrinsic factors has been suggested; indeed more than 60 % of BD patients are associated with HLA-B51 [5] and some cases may be also associated with HLA-B52 [6].

Sensorineural hearing loss and vertigo have been described in adult patients with BD, but are exceptionally rare in paediatric age [7].

We report the case of a 15 year-old boy who had recurrent episodes of vertigo and hearing loss that was finally diagnosed with a BD. All the symptoms resolved with the administration of the anti-tumor necrosis factor (TNF)-α drug adalimumab.

## Case presentation

A 15 year-old boy of Italian descent presented to our Department with a six-month history of recurrent episodes of fever, vomiting, dizziness, headache, tinnitus and ataxia. He had no significant past medical or family history. Magnetic resonance imaging (MRI) performed on day 7 post onset of symptoms excluded cerebral and brainstem lesions. A vestibular viral neuritis was diagnosed and the boy was continuously treated with oral steroids with partial improvement.

On admission the boy was conscious, his body temperature was 37°C and blood pressure was within normal limits (100/60 mm Hg). He had dizziness, horizontal nystagmus which was aggravated by head movements. He was not able to sit or stand up. Romberg’s sign and head-thrust test were positive. No visual problems, meningeal signs, clonus or pathologic reflexes were noted. Both tympanic membranes were normal.

Laboratory tests resulted normal except for a slight elevation of erythrocyte sedimentation rate (ESR: 40 mm/h) and C-reactive protein (CRP: 4.5 mg/dl); a neutrophilic leucocytosis was noted. Cerebrospinal fluid (CSF) analysis revealed 3 nucleated cells/field, normal glucose and protein levels and negative tests for Herpes virus 1-2, Enterovirus and Borrelia Burgdorferi. Cultures of CSF, blood, urine and throat swabs showed negative results. Varicella-zoster, Borrelia Burgdorferi, Cytomegalovirus and Epstein-Barr virus serology were also negative. Anti-cochlear and anti-nuclear antibodies (ANA) were absent. The haplotype HLAB52 was found.

Audiometry revealed bilateral sensorineural hearing loss. Chest x-ray, abdomen ultrasound and brain MRI excluded any abnormality. An electroencephalogram showed normal results.

An autoimmune audio-vestibular disease was suspected, the boy was treated with methylprednisolone (2 mg/Kg/day). After clinical improvement, a few weeks later, steroid therapy was gradually tapered and discontinued.

Two months later, however, febrile episodes recurred with associated oral ulcers, dizziness and tinnitus. At that time, eye examination showed anterior uveitis and retinitis in the right eye.

Colonoscopy and esophagogastroduodenoscopy were normal whereas endoscopic video capsule showed a few ulcers surrounded by healthy mucosa in jejunum and ileum. Abdominal MRI, however, excluded intestinal Crohn's disease.

The association of oral ulcers, uveitis, recurrent fever and neuro-vestibular symptoms suggested BD. The patient was started on intra-ocular steroids, oral colchicine 1 mg/day and continued low-dose oral prednisone.

Three months later, however, a new flare up of disease was observed. So, the boy was started with adalimumab at 24 mg/m^2^ subcutaneously every other week. His symptoms completely resolved soon after the therapy was introduced and prednisone was discontinued two months later.

Follow-up audiometry, laboratory evaluation and ocular screening recorded normal values.

At two-year follow-up, the boy continues adalimumab treatment and is symptom free.

### Case discussion

To the best of our knowledge, this is the first case described of paediatric BD with audio-vestibular manifestations responding to adalimumab. Our patient initially presented vertigo, fever and hearing loss, symptoms that were difficult to classify at a first examination.

Differential diagnosis included a large group of syndromes characterized by dysfunction of the inner ear causing vertigo and hearing loss (Table 1).

AIED accounts for less than 1 % of all cases of hearing loss; it has an immune-mediated pathogenesis involving both innate and adaptive immune system, developing an immune reaction in the endolymphatic sac, ultimately leading to the destruction of sensory and supporting cells within the cochlea. Cytokines such as IL-1, IL-2 and TNF-α and the transcriptor factor NF-kB are involved in the immune response [1]. Given the absence of specific tests, the diagnosis of AIED may be difficult and is mainly based on the association of appropriate clinical presentation (bilateral hearing loss with associated vertigo in about 50 % of cases), exclusion of other causes, and a positive response to steroid treatment [1].

In every patient who has progressive bilateral sensorineural hearing loss with no other explainable cause, AIED should be considered, a systemic autoimmune disease associated should be investigated and specific hematologic tests (complete blood count, ESR, CRP, ANA e anti-cochlear antibodies, C3 and C4 complement levels) should be performed. After, a trial with steroids (1mg/Kg/day of prednisone or methylprednisolone) should be attempted. However, the overall response rate to steroids is about 50-70 % of cases; in patients non-responders to corticosteroids, immunomodulatory agents such as methotrexate and anti-TNF-α drugs (adalimumab, etanercept and infliximab) can be used [1].

In our patient, diagnosis was delayed and was possible only when the classic symptoms of BD disease appeared and one could assume that the ongoing steroid therapy contributed to delay the onset of symptoms and therefore the correct diagnosis.

The diagnostic criteria for BD were defined by the International Study Group (ISG) for BD in 1990, and include the presence of recurrent oral ulceration, with at least three episodes over 12 months, in addition to two of the following features: recurrent genital ulcers, eye lesions, skin lesions and a positive pathergy test [8]. Recently new criteria have been developed in order to facilitate the diagnosis both in adults and in paediatric patients [2, 9]. Indeed our patient didn’t fulfil the ISG criteria, whereas he presented the criteria for the diagnosis of paediatric BD (neurological signs, recurrent oral aphthosis and ocular involvement) [9]. In addition he carried the allele HLA-B52, an antigen that has also been associated with BD [6].

BD treatment includes colchicine, corticosteroids, dapsone and immunosuppressive drugs in severe cases. The choice of therapy, however, is limited by the scarceness of high-quality therapy trials and is based largely on case reports, case series and few randomized clinical trials [10].

High levels of TNF-α and its soluble receptor have been found in the serum and aqueous humor of patients with active BD [11]. For this reason, anti-TNF-α treatment has been increasingly reported in BD, especially in refractory severe disease [12, 13]. Our patient presented a rapid and sustained response to adalimumab treatment. Complete resolution of audio-vestibular symptoms, ocular inflammation and oral ulcerations was noted; furthermore febrile attacks subsided as soon as adalimumab treatment was started. Besides, no adverse events were observed in two years follow-up.

## Conclusions

This case report points out to the diagnostic and therapeutic challenges of BD especially when unusual symptoms are the prominent manifestations of the disease. It also suggests that adalimumab is a good therapeutic option in children with BD and audiovestibular symptoms.

## Abbreviations

AIED: Autoimmune inner ear disease
BD: Behçet disease
CRP: C-reactive protein
ESR: Erythrocyte sedimentation rate
ISG: International study group
MRI: Magnetic resonance imaging
TNF-alfa: Tumor necrosis factor-alfa
